# Pathogens, Commensals, and Immunity: From the Perspective of the Urinary Bladder

**DOI:** 10.3390/pathogens5010005

**Published:** 2016-01-07

**Authors:** Bruce Beutler

**Affiliations:** Center for the Genetics of Host Defense, The University of Texas Southwestern Medical Center, Dallas, TX 75390, USA; bruce.beutler@utsouthwestern.edu

Why study immunity as it pertains to the urinary bladder? As it happens, the bladder is a wonderful model to analyze, one of the best available for understanding regulation of immune responses to commensals and pathogens. However, some preamble is needed to explain why. We might start by considering basic parameters, such as the general relationship between the immune system, pathogens, and commensals, and how *Homo sapiens*, one species among all the millions that evolved immunity, deals with microbes.

The battle between host and pathogen is ultimately a battle of genomes. The genome of the host spawns an immune system, which may or may not be able to defeat a particular pathogen, sterilizing or at least containing it. The pathogen genome, for its part, spawns an aggressive set of molecular tools used to invade the host, harvest its resources, proliferate, thwart immunity, and transmit its descendants to another host. 

The battle was once waged without much conscious effort on the part of either participant. Nowadays, that has all changed. The human brain, the ultimate artifice of the genome (one about which we understand much less than the immune system) has emerged as a powerful defense system in its own right. There’s no denying this, or dismissing it as a semantic argument. The human brain is capable of anticipating pathogens, actively avoiding them (by directing us to boil drinking water, for example), vaccinating against them, and designing and mass producing chemotherapeutics to eliminate them if they cause an infection. The “collective” human brain understands all classes of pathogens in considerable detail, is constantly learning more about them and their vulnerabilities, and is quick to recognize new ones whenever they arise. The brain has even developed its own set of adjunctive tools—computers—that enormously assist it in visualizing the molecules of microbes, solving their genomic sequences, and predicting the structures and functions of all of their proteins.

When all is said and done, the brain can hardly substitute for an immune system, except insofar as we might manufacture a sterile world for ourselves, and derive our species to live within it. However, the brain has thrown enormous weight into the battle, perhaps increasing our resistance to infection by a factor of ten or more. It might be said to synergize with the immune system, since for most of us, neither the brain nor the immune system acting alone would suffice to give long life. Certainly medical prevention and intervention saves many lives that would otherwise be lost prematurely to infectious disease. In developed countries of the world, *most* lives are saved by the brain and its creations, and death from infection is quite rare as a result… at least for now. In less developed nations, infection continues to be the major cause of human death.

A point of particular interest concerns the distinction between those microbes that are harmful and those that are not: that is to say, the difference between pathogens and commensals. The principal difference between a pathogen and a commensal is that the latter does not encode such aggressive tools for invasion. The commensal’s strategy does not involve aggression. The host’s strategy is, more or less, to ignore the commensal. This is not to say that that a commensal can never be harmful, and the distinction does become blurry. Absent an immune system, most commensals would need to be redefined as pathogens. According to our current understanding, commensals are even to be welcomed, because while they don’t direct their aggression against us, they may direct it against pathogens, with which they compete for niches in the extracellular milieu. Certainly, they may also prime the immune system for action against pathogens.

It is a curious fact that the immune system, like the brain, can distinguish commensals from pathogens. Moreover, the brain, as yet, remains somewhat puzzled as to how the immune system accomplishes this. For example, both pathogenic *E. coli* and commensal *E. coli* may produce equivalent amounts of chemically identical LPS, a quintessential innate immune activator that engages a Toll-like receptor, TLR4. Yet we respond to the pathogen and not to the commensal. Sometimes this may reflect the fact that a pathogen breaches epithelial barriers, but not always, and perhaps most of the time something more subtle occurs. In fact, this is the key question that the urinary bladder model may allow us to answer.

Some hypothesize that damage caused by microbes, rather than molecules of microbes per se, is the prime activator of an immune response. In certain situations, this may indeed be the case. However, if so, we still don’t have a complete picture of the molecular events that sound an alarm most of the time, summoning first innate immune cells, and then prompting an adaptive immune response, where before all was quiet. We might guess, too, that once the alarm is sounded, a good deal of tissue damage results. Perhaps as much damage is caused by the immune response itself as by the action of the pathogenic microbes. At that point, damage to barrier tissues can, by itself, permit microbes to enter the host, including microbes that weren’t pathogens to begin with. Some commensals may then manifest pathogenic effects, even if their genetic program didn’t originally call for it.

It is speculated that diseases like ulcerative colitis may result simply because the immune system has a problem distinguishing commensals from pathogens, and “overreacts.” In this view, inflammatory bowel disease is caused by a failure of normal immune homeostatic mechanisms. So the question of how the immune system sets a threshold for its response is an important one. Moreover, the question isn’t confined to the colon, but applies elsewhere as well. Discrimination between commensals and pathogens, however it occurs, is exercised at almost every boundary between the host and the world beyond: within the GI tract, the upper and lower airways, the genital epithelium, the surface of the skin, and the surface of the eyes, for example. In the context of the present discussion, we must consider how discrimination occurs within the urinary bladder.

Asymptomatic bacteriuria (ABU) is quite common, and at some level, may be almost universal. This we may see as a form of commensalism. The initial question to ask would be: is it purely the microbe that determines whether a given host will have ABU as opposed to bladder infection or pyelonephritis? Apparently it is not. Host factors seem to be equally important, and perhaps more important, as determinants of pathology (though most certainly, there are also virulence factors associated with microbes that cause disease).

The host side of the equation is particularly important for us to understand. The microbial world changes continuously, and the future may see the emergence of many different strains and virulence mechanisms. In a way, we have less control over the microbial world than we do over ourselves. On the host side, things evolve much more slowly. We need to study host susceptibility from every angle. Could host anatomical differences have key importance? Are there genetic predictors of infection *vs.* ABU, and can we, in the post-genome era, track these determinants down? What are the molecular details of pathogenesis, and can tools of cell biology and immunology help us to understand how disease develops? These are among the major challenges researchers in this field must confront.

The bladder offers a much simpler model of interactions between host and microbe at the epithelial level as compared to the colon, skin, or other barrier tissues. The microbial flora is less complex (urine is sometimes truly sterile, or has a low content of microbes). The flora can sometimes be fully defined by sequencing. Abundant clinical material is available for study, it is possible to induce experimental infections with relatively low risk, and infections can be monitored by cystoscopy and/or urine collection. As models go, there are few that are better. Yet animal models, particularly mouse models, will certainly also have their place in understanding host microbe interactions in the bladder.

The second Molecular UTI Conference in Malmö, held 25–27 August 2014, was one of the devices the brain has come to use to solve complex problems in immunity: bring together a collection of experts studying diverse aspects of the problem, and let them discuss the big picture freely and amicably. The topics covered included all of those mentioned above, and quite a few others besides, bearing on the metabolism of bacteria, drug therapy, the exact way in which bacteria elicit damage and pain when the bladder is infected, and the nature of the “conversation” between host and pathogen, *vs.* host and commensal. It was a rewarding experience, and the following compendium of papers will surely spur progress in this important field.

